# Microbial Biodiversity in Sediment from the Amuyo Ponds: Three Andean Hydrothermal Lagoons in Northern Chile

**DOI:** 10.3390/microorganisms12112238

**Published:** 2024-11-05

**Authors:** Claudia Vilo, Francisca Fábrega, Víctor L. Campos, Benito Gómez-Silva

**Affiliations:** 1 Laboratory of Biochemistry, Biomedical Department, Health Sciences Faculty, Centre for Biotechnology and Bioengineering (CeBiB), Universidad de Antofagasta, Antofagasta 1240000, Chile; claudia.vilo@uantof.cl (C.V.); francisca.fabrega@uantof.cl (F.F.); 2 Health Sciences Faculty, Universidad del Alba, Santiago 8320000, Chile; 3 Graduate Program in Applied Sciences: Aquatic Systems, Universidad de Antofagasta, Antofagasta 1240000, Chile; 4 Laboratory of Environmental Microbiology, Department of Microbiology, Faculty of Biological Sciences, Universidad de Concepcion, Concepcion 4070386, Chile; vcampos@udec.cl

**Keywords:** Amuyo Ponds, Atacama Desert, biodiversity, extreme environments, metagenomics, microbial and viral communities

## Abstract

The Amuyo Ponds (APs) are a group of three brackish hydrothermal lagoons located at 3700 m above sea level in a pre-Andean setting in the Atacama Desert. Each pond shows a conspicuous green (GP), red (RP), or yellow (YP) coloration, and discharges water rich in arsenic and boron into the Caritaya River (Camarones Basin, northern Chile). Microorganisms are subjected to harsh environmental conditions in these ponds, and the microbial composition and diversity in the Amuyo Ponds’ sediments are unknown. The microbial life colonizing AP sediments was explored by metagenomics analyses, showing a diverse microbial life dominated by members of the bacterial domain, with nearly 800 bacterial genome sequences, and sequences associated with Archaea, Eukarya, and viruses. The genus *Pseudomonas* was more abundant in GP and YP sediments, while the genera *Pseudomonas*, *Aeromonas*, and *Shewanella* were enriched in RP sediments. Archaeal composition was similar in all sediments, and enriched with methanogens sequences from the *Archaeoglobi* and *Halobacteria* classes. Abundant fungi sequences were detected in all sediments from the phyla *Blastocladiomycota* and *Ascomycota*. We also report putative functional capabilities related to virulence and defense genes, the biosynthesis of secondary metabolites, and tolerance to arsenic. Thirteen bacterial and fourteen viral metagenome-assembled genomes were reconstructed and informed here. This work expands our knowledge on the richness of the microorganisms in the APs and open further studies on the ecology and genomics of this striking Andean geosite.

## 1. Introduction

Metagenomics studies are culture-independent approaches that improve our understanding on the identification, composition, structure, and genomics of microbial communities in sediments associated with natural or contaminated water bodies. Also, metagenomics is key to gaining insights on the roles of microorganisms in ecosystems, their adaptive strategies, and their metabolic and functional capabilities to colonize environments under extreme physical and chemical conditions; it also allows us to acquire useful genetic information on members of these microbial communities, new genetic resources, and biosynthetic pathways, and may allow genome reconstructions [1,2]. Particularly, metagenomic sequencing has improved our knowledge on taxonomic diversity, metabolic potential, and the roles of microbial communities present in various hydrothermal sediments [3,4,5,6].

The Atacama Desert extends across 1000 km, from 30° S to 20° S along the Pacific coast of South America, and it is known as a territory under polyextreme environmental conditions, where life is mostly restricted by two major natural factors: desiccation and solar radiation [7]; however, this dryland is a biodiversity hotspot with genetic richness that includes prokaryotic and eukaryotic life forms adapted to life-limiting environmental conditions [7,8,9]. Rivers, lakes, and ponds in the Atacama Desert, where water is not a limiting factor, are proper habitats for life; most of these water bodies are found in the pre-Andean and Andean territories, where precipitations are 50-fold higher than those in Atacama’s hyper-arid core [10]. In these territories, the water chemistry of surface and ground waters is influenced by active and regular volcanism, weather regimes, and water–rock–soil interactions [11,12,13,14,15,16,17]. Under such influence, the hydrochemistry of water bodies impacts on the abundance and diversity of microbial communities, favoring the colonization of microbes with adequate adaptive solutions.

The Amuyo Ponds (AP) in northern Chile are an example of such a situation. They are three colored ponds, the Yellow Pond (YP), Red Pond (RP), and Green Pond (GP), located at 3700 m above sea level in the Arica and Parinacota Region in northern Chile. They have geothermal origins, and their transparent and brackish waters are rich in arsenic, boron, and other dissolved salts, and drain into the Caritaya River, a main affluent for the Caritaya Dam within the Camarones Basin [11,14,17]. Their characteristic colorations are provided by mineral precipitations (carbonate, sulfate, silica) forming dome-like structures and rendering colors to the crusts and sediments of each pond. Hematite and realgar (As_4_S_4_) and calcite and orpiment (As_2_S_3_) have been identified in the Red Pond and Yellow Pond, respectively; the presence of other arsenic sulfide minerals has not been disregarded, and the probable source for the coloration of the Green Pond has not been reported [17]. Substantial hydrogeochemical studies have been conducted on the APs, but limited biological information is available. Previous reports showed the presence of amphipods and microbial mats in small saline pools around the ponds [17], and bacteria have also been isolated to evaluate their agricultural potential [18]. Thus, the APs are geothermal habitats colonized by a partially known microbiome enduring the prevailing physical and chemical environmental conditions, e.g., high solar radiation, diel temperature changes, and high boron and arsenic concentrations, as found in Laguna Tebenquiche [5] and other aquatic habitats. AP sediments appear to be a more challenging environment for microbial life than Amuyo waters, based on substantial differences in their chemical composition [16,17,18]; substantial differences are also found among the sediments of each pond. Here, we report the first metagenomics insight on the microbial composition, diversity, and differences among AP sediments, showing that microbial life in these ponds is dominated by members of the bacteria domain, with a much lower presence of members from the Archaea and Eukarya domains, and a large viral presence and diversity. We also report examples of putative functional capabilities and metagenome-assembled genomes (MAGs) of select microorganisms and viruses. Preliminary reports on metagenomics analyses of the Amuyo Ponds’ sediments and metal resistance were presented at the XIV Workshop CeBiB: Genomics, Bioinformatics and Metabolic Engineering for Biotechnological Applications, during 6–8 December 2021 at Santa Cruz, Chile, and 29–30 November 2022, at Santiago, Chile.

## 2. Materials and Methods

**Figure 1:**
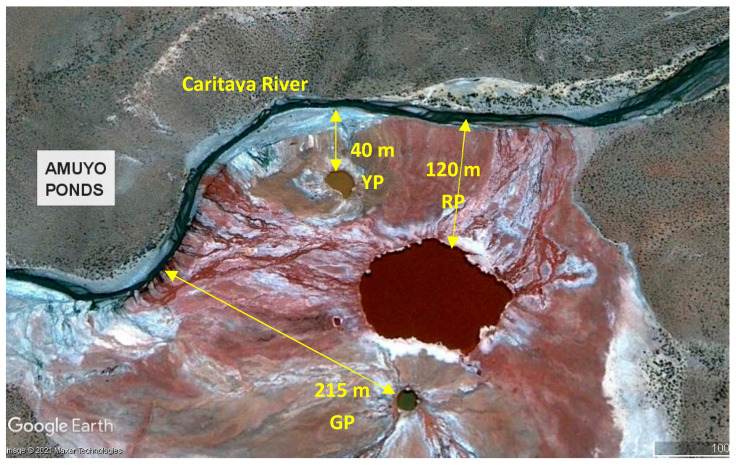
Proximity of Amuyo Ponds to Caritaya River, Camarones Basin, Region of Arica and Parinacota in northern Chile. YP, RP, and GP correspond to yellow, red, and green ponds, respectively. (Image obtained from Google Earth Pro, accessed in April 2024).

**Figure 2:**
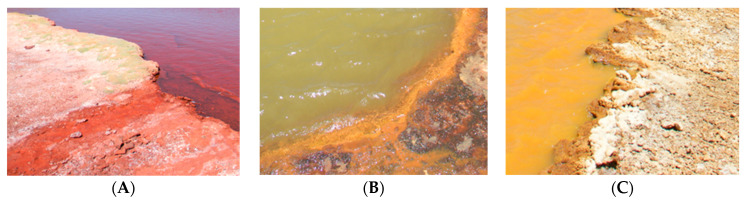
View of sediments at the edge of each Amuyo pond ((**A**): Red Pond; (**B**): Green Pond; (**C**): Yellow Pond).

***Site description and sampling***. Yellow Pond (19°03′25″ S, 69°15′12″ W), Red Pond (19°03′28″ S, 69°15′10″ W), and Green Pond (19°03′31″ S, 69°15′11″ W) are three hydrothermal colored ponds located at 3700 m above sea level in the Arica and Parinacota Region in northern Chile. Based on images from Google Earth Pro (accessed and analyzed in October, 2021), the closest distances of YP, RP, and GP to the Caritaya River margin are 40 m, 120 m, and 215 m, respectively, and the closest distances between them are 40 m (RP to GP), 60 m (RP to YP), and 175 m (YP to GP) (Figure 1). Sediment samples were aseptically obtained from the surface to 5 cm depth at the edge of each pond. From each pond, three samples (10–15 g each, approximately) were collected at three different, accessible and safe sites (5–10 m apart) and kept at 4 °C during transportation. At the laboratory, each group of sub-samples was combined into a single composite sample to obtain a single representative sediment sample per pond. These were maintained at 4 °C until use (Figure 2).

***DNA extraction, sequencing, and metagenomics analyses***. Total DNA from the upper 2 cm sediment layer (1 g) was extracted using the Ultraclean DNA extraction kit (MO BIO Laboratories Inc., Carlsbad, CA, USA), following the protocol provided by the manufacturer. Quality and DNA concentrations were evaluated by gel electrophoresis and UV/Vis spectroscopy (NanoDrop ND-1000, Peq-lab, Erlangen, Germany). The total genomic DNA extracted, including all members of each Amuyo Pond’s microbial community, was submitted for sequencing analysis at the Greehey Children’s Cancer Research Institute Next-Generation Sequencing Facility, UT Health Science Center, University of Texas System, San Antonio, TX, USA. Sequencing was achieved using Illumina HiSeq technology with paired-end reads and an average read length of 100 bp, with good quality scores, as evaluated by the FastQC program (version 0.10.0). The sequencing produced a total of 76,170,294 reads after QC. The sequencing reads are available at the Sequence Read Archive (SRA) with accession numbers SRR30290574; SRR30505203; and SRR30505202.

***Bioinformatic Analysis***. The metagenomic sequences were submitted to the Rapid Annotation using Subsystems Technology for Metagenomes (MG-RAST) web server [19] for a taxonomic and functional assignment using default parameters. In addition, metagenomic assembly was performed using MEGAHIT assembler v.1.2.9 [20], and binning was conducted using the PATRIC web server [21]. The complete genome was annotated using the Rapid Annotations using Subsystem Technology (RAST) server version 4.0. In addition, in-house BLAST analysis was performed against customized metal resistance genes databases.

## 3. Results

### 3.1. Metagenomic Sequence Statistics and Relative Abundance of Microbial Domains

Comparative statistics of the sequences post-QC are shown in Table 1. GP, YP, and RP sediments accounted for nearly 23, 25.5, and 27.5 million sequences, respectively. Sequences from the GP and RP sediments showed similar GC% (57%), while a higher percentage was observed in YP sediment (62%). The post-QC sediment sequences were dominated by bacterial sequences in all Amuyo Ponds, providing an insight on their microbial community composition and diversity (Table 2). Other minor sequences were related to viruses and the Eukarya and Archaea domains.

### 3.2. Relative Abundance of Bacterial Classes, Phyla, and Genera in Amuyo Ponds’ Sediments

**Figure 3:**
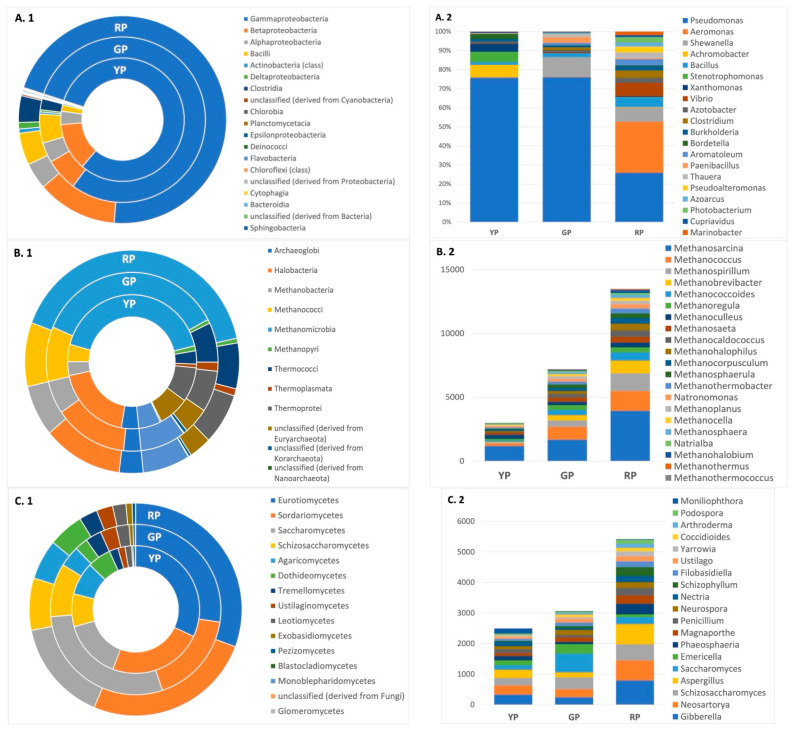
Relative abundance of classes (**A.1**,**B.1**,**C.1**) and genera (**A.2**,**B.2**,**C.2**) for bacteria, Archaea, and fungi, respectively, in sediments from Amuyo Ponds.

After metagenomics analyses, the relative abundance for major bacterial classes in Amuyo sediments was as follows: *Gammaproteobacteria* > *Betaproteobacteria* > *Alphaproteobacteria* > *Bacilli*. The genus *Pseudomonas* was more abundant in GP and YP sediments, while the genera *Pseudomonas*, *Aeromonas*, and *Shewanella* were enriched in RP sediments (Figure 3). Archaeal sequences were more abundant in RP sediments, but all three sediment types showed similar composition and were enriched with sequences belonging to the classes *Archaeoglobi* (phylum *Euryarchaeota*) and *Halobacteria* (phylum *Euryarchaeota*) (Figure 3). Also, the most abundant Fungi sequences in the Amuyo sediments belonged to the classes *Blastocladiomycetes* (phylum *Blastocladiomycota*), *Eurotiomycetes* (phylum *Ascomycota*), and *Saccharomycetes* (phylum *Ascomycota*), with an interesting genus diversity (*Gibberella*, *Neosartoria*, and *Scizosaccharomices*) among them (Figure 3).

### 3.3. Relative Abundance of Archaea and Fungi Among Amuyo Ponds’ Sediments

The archaeal community in AP sediments was found to be enriched in methanogens (Figure 3(B.2)). The genus *Methanosarcina* (*Euryarcheota* phylum) is a highly efficient methane producer, and was most abundant, suggesting that members of the Amuyo microbial community may be considered for new models to inquire into methanogenesis and methanotrophic cycles in such environments. Also, the metagenomic sequences retrieved from the AP sediments showed diversity and major relative abundance in the fungal genera *Gibberella*, *Neosartorya*, *Schizosaccharomyces*, *Aspergillus*, and *Saccharomyces* in all three ponds’ sediments (Figure 3(C.2)).

### 3.4. Relative Abundance and Distribution of Bacterial Genera Among Amuyo Ponds’ Sediments

**Figure 4:**
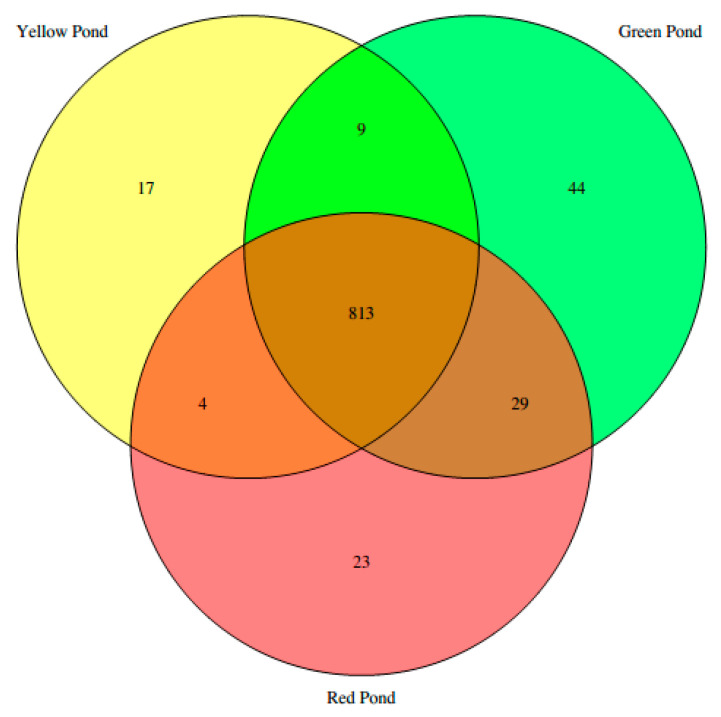
Venn diagram showing the distribution of bacterial genera in Amuyo Ponds’ sediments. A total of 813 genera were detected by metagenomic analyses, while 17, 44, and 23 genera were assigned to Yellow, Green, and Red Ponds, respectively. The numbers of shared bacterial genera were 4, 9, and 29 between the Yellow and Red Ponds, Yellow and Green Ponds, and Green and Red Ponds, respectively.

The substantial number of microbial sequences obtained from the metagenomic analyses of AP sediments and the relatively close proximity among Amuyo Ponds afford an insight into the microbial distribution similarities and differences among the three ponds (Figure 4). More than 800 bacterial genera were shared among the AP sediments, but a lower, distinct number can be assigned to each pond: 17, 23, and 44 genera for YP, RP, and GP, respectively. Additionally, 4, 9, and 29 bacterial genera were found to be shared between sediments from YP and RP, YP and GP, and GP and RP, respectively.

### 3.5. Metavirome in Amuyo Ponds’ Sediments

**Figure 5:**
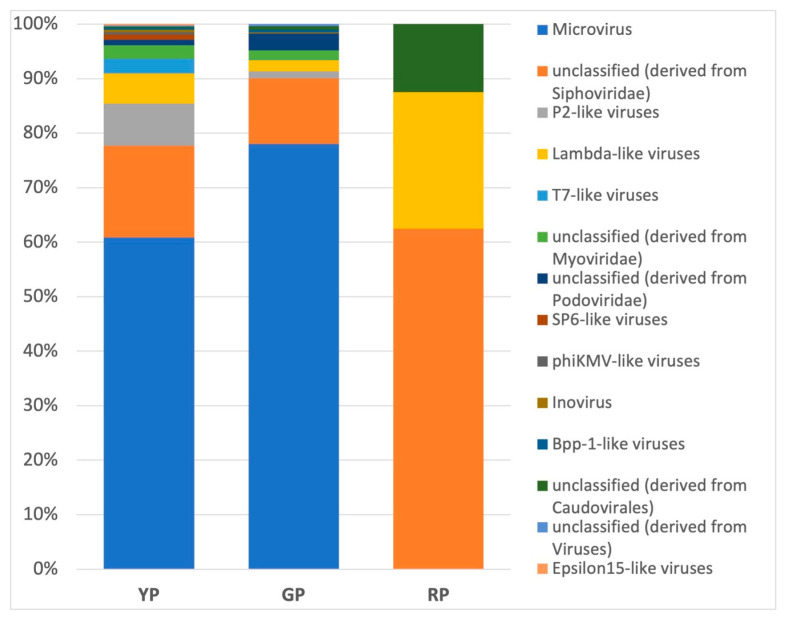
Relative abundance of virus genera in sediments from Amuyo Ponds.

A diverse set of metagenomics sequences related to the AP sediments’ metavirome is shown in Figure 5. Unclassified phages derived from the *Siphoviridae* family were most abundant in the YP and GP sediments, while P2-like viruses (family *Myoviridae*) were enriched in RP sediments. This is a first insight on viruses from these conspicuous hydrothermal habitats and raises questions on their molecular structure and on the interaction between Amuyo viruses and their hosts.

### 3.6. An Insight on the Metabolic Roles of Members of the Microbial Community

**Figure 6:**
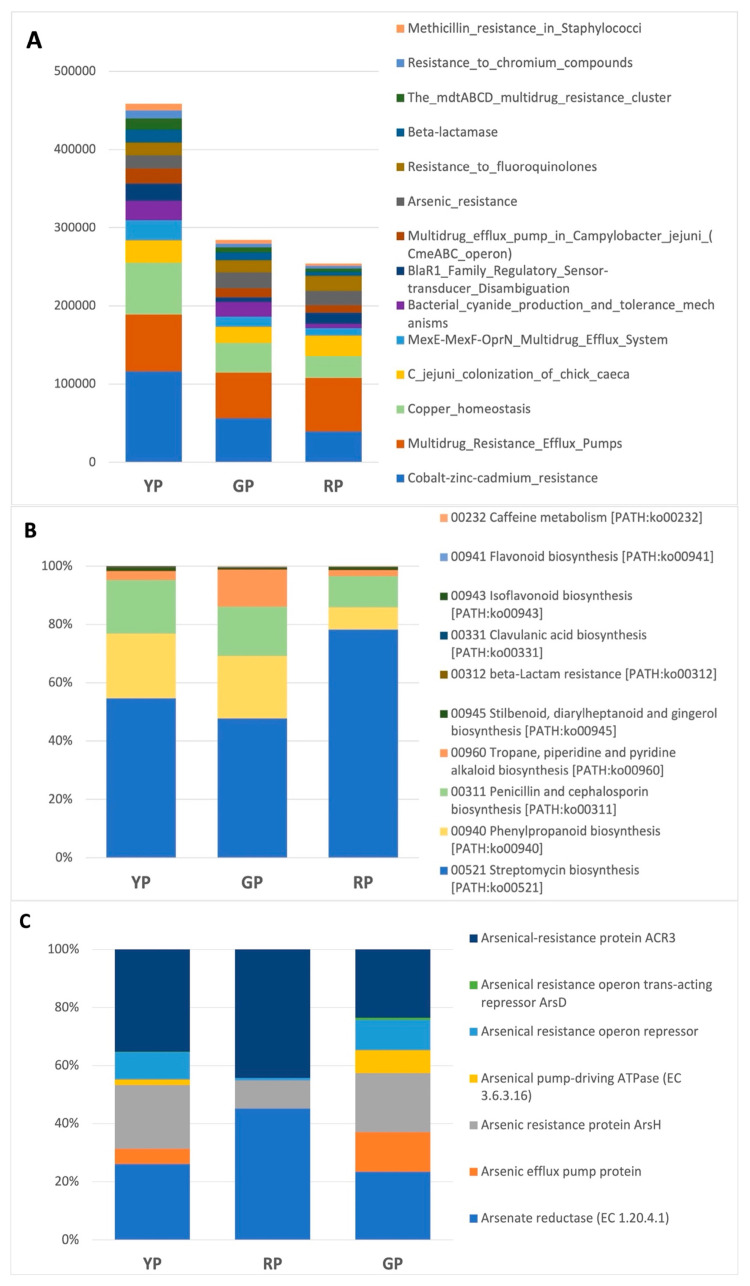
Putative bacterial genes in Amuyo Ponds’ sediments. (**A**): virulence and defense; (**B**): biosynthesis of secondary metabolites; and (**C**): arsenic resistance. Y axis indicates absolute abundance in (**A**) and relative abundance in (**B**,**C**).

**Figure 7:**
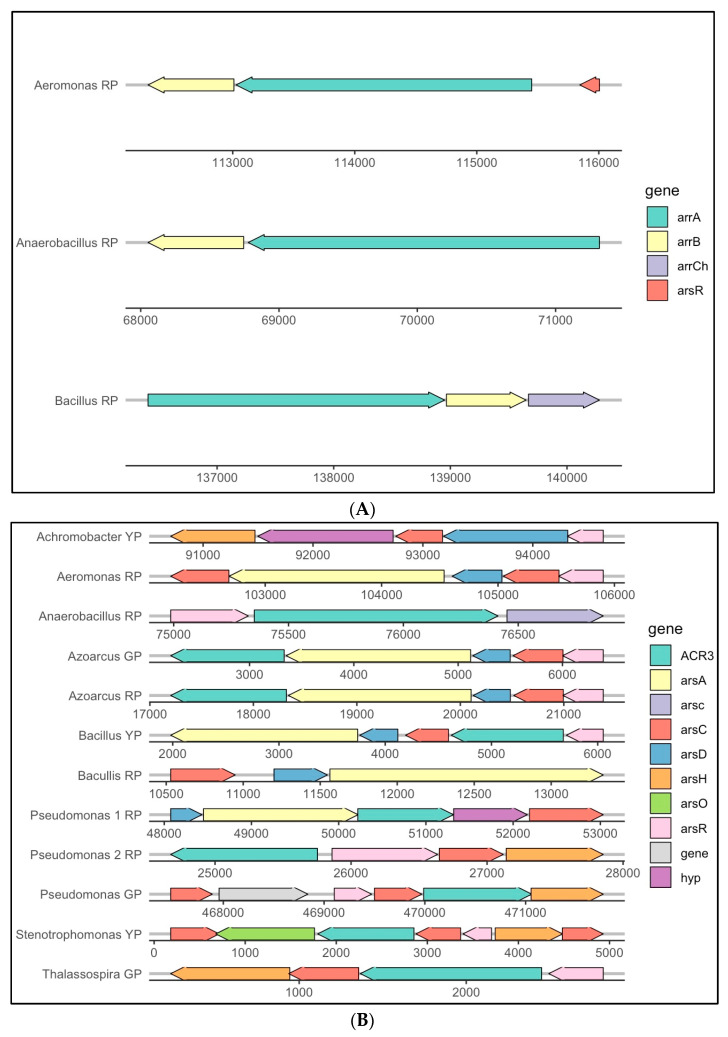

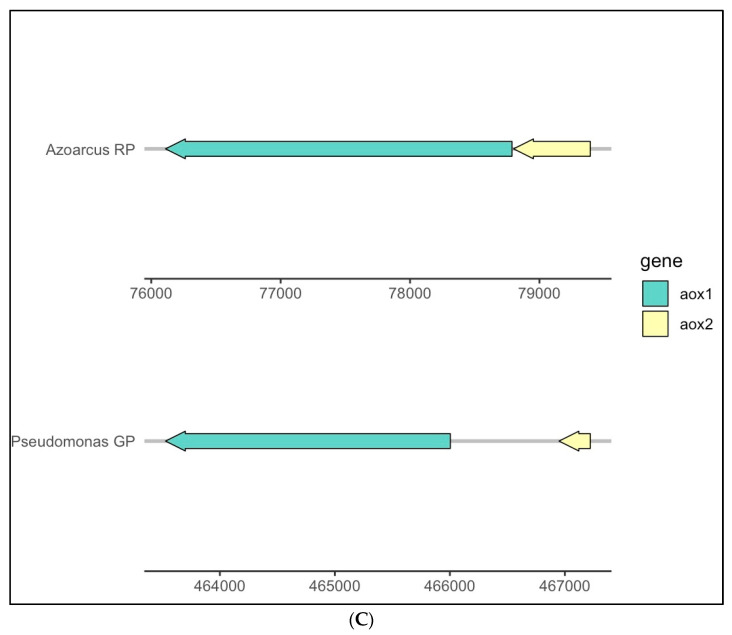
Putative bacterial gene clusters associated with arsenic resistance capabilities in metagenomic-assembled genomes from Amuyo Ponds’ sediments. (**A**): arsenic reductase bacterial gene clusters; (**B**): arsenic respiratory reductase gene cluster in Red Pond; (**C**): arsenite oxidase gene cluster.

Putative metabolic capabilities were inferred from the Amuyo sediment sequences (Figure 6). RP sediments rendered a higher number of predicted functions, most probably due to the larger post-QC sequences. As an example, a partial list of putative genes related to virulence and defense genes and the biosynthesis of secondary metabolites is shown in Figure 6A,B. Among them, genes associated with metal resistance (Zn, Hg, Co, Cd), fluoroquinolones, and copper homeostasis were observed (Figure 6A). Putative genes for the biosynthesis of streptomycin, penicillin, and cephalosporin were also detected (Figure 6B), as well as genes related to arsenic resistance (Figure 6C). Further gene mining using reconstructed genomes allowed us to demonstrate the presence of gene clusters associated with arsenic reductase in bacterial members from all three Amuyo Ponds (Figure 7A). Arsenic respiratory reductase was only found in *Aeromonas*, *Anaerobacillus*, and *Bacillus* members in Red Pond sediments (Figure 7B), and the arsenite oxidase gene cluster was found in *Azoarcus* and *Pseudomonas* genomes from the Red and Green ponds, respectively (Figure 7C).

### 3.7. Metagenome-Assembled Genomes (MAGs) in Amuyo Sediments

Table 3 shows thirteen constructed bacterial MAGs with completeness and error percentages. The *Pseudomonas* MAG was obtained from sequences retrieved from the three Amuyo sediments. The assembly of MAGs for *Stenotrophomonas*, *Achromobacter*, and *Thalassospira* was achieved with sequences from the YP sediments. The *Azoarcus* MAG was reconstructed from sequences obtained from the GP and RP sediments. The *Bacillus* MAG was prepared with sequences from the YP and RP, while sequences for the *Anaerobacillus* MAG and *Aeromonas* MAG came from RP and GP sediments, respectively. Most reconstructed bacterial MAGs showed over 95% completion with less than 5% error.

Also, fourteen viral MAGs were assembled from the Amuyo sediment sequences, with the corresponding completeness and error percentages shown in Table 4. Among them, the MAGs for *Bacillus* phage in YP sediments, Bacteriophage Lily plus *Rhizobium* phage RR1-B in GP sediments, and Circular genetic element sp. in RP sediments were identified. Viral MAGs showed over 90% completion with less than 10% error.

## 4. Discussion

Metagenomics as a culture-independent approach provides evidence on the presence and partial identification of life forms in their habitats; also, metagenomics contributes to information on new genetic resources, adaptive strategies, functional capabilities, and biosynthetic pathways, and may allow genome reconstructions [1,2,5,22].

***On Amuyo Ponds’ microbiome***. The presence of a microbiota containing diverse extremophiles and extremely tolerant microorganisms has been extensively reported in the last two decades in various geosites of the hyper-arid Atacama Desert, including salars, lakes, geysers, volcanoes, and geothermal aquatic systems at high altitude in the Andes Mountains under geological, hydrochemical, climatic, and/or volcanic influences [7,12,18,22,23,24,25,26,27,28,29,30]. The Amuyo Ponds are geothermal habitats colonized by a partially explored microbiome enduring the local and prevailing abiotic environmental conditions. Recently, fifteen isolates were recovered from AP sediment samples: fourteen were assigned to the *Proteobacteria* phylum (twelve to the *Gammaproteobacteria* and two to *Alphaproteobacteria* class) and one to the *Bacillota* phylum (*Bacilli*), although their pond origins were not clearly annotated [18]. We have identified *Proteobacteria* as the most abundant bacterial phylum in AP sediments by a culture-independent approach.

The APs contain brackish waters with Na^+^ and Cl^−^ as dominant ions, with an average concentration of 0.3–0.4% *w*/*v*, a stable pH (7.3–7.7), and surface water temperatures of 25–33 °C. Only the RP shows the highest maximum depth temperature of 57 °C. One major characteristic of the APs is their high arsenic and boron contents [13,14,15,16,17,18]. Then, it is reasonable to infer that the APs’ microbial members are not subjected to high salinity or extremes temperatures or pH, and do not need adaptive mechanisms to overcome such stressful conditions. However, differences in microbial composition among these ponds is another observation from our metagenomic work: sediments from the YP and GP were enriched in Gammaproteobacteria, with *Pseudomonas* as the major genus, while the genera *Aeromonas* and *Shewanella* were abundant in RP sediments; additionally, each pond sediment contains different and unique groups of bacterial genera, but the GP sediment has the largest number of unique bacterial genera (Figure 4). The reason for such difference is an open question, but we hypothesize that dissimilarities in the chemical composition of their sediments, rather than other abiotic factors, are the driving force influencing microbial composition. This explanation is partially inferred from the analyses conducted previously in AP waters and sediments [13,14,15,16,17,18]. It is worth mentioning here that seasonal differences were observed in the environmental variables measured in the APs during winter [14] and late summer [18], and may also have an impact on the APs’ microbiome composition.

From the chemical data reported by Muñoz-Torres et al. [18] on AP sediments, it can be inferred that (a) all three sediment types have similar contents of boron, arsenic, Cu, Mn, Zn, and K; (b) the highest concentrations of Ca, Mg, Fe, sulfate, nitrate, carbonate, and bicarbonate were found in GP sediments; (c) the phosphate content was 14 times and 2 times higher in RP and GP sediments (respectively) than in YP sediments; (d) comparatively, nitrate (0–4 mg L^−1^) and phosphate (5–70 mg L^−1^) were in limited supply in the AP waters and sediments; (e) all AP sediments showed substantially greater total arsenic, Fe, Zn, Mn, and Cu concentrations than their corresponding waters, with differences in contents among the sediments; and (f) the electric conductivity levels in the waters and sediments from the RP and YP were similar (12–16 mS cm^−1^). The conductivity of the GP sediments was double the conductivity of the GP water; also, all ponds have reductive lower sediments [13,14,15,16,17,18]. Thus, these and probably other still unidentified metals and metalloids may influence the differences in microbial abundance, diversity, and composition among the AP sediments.

***Microbiome of other Andean hydrothermal sites***. Other geosites, like the Andean Lirima hydrothermal ecosystem, also contain reductive sediments (−250 to −3020 mV), similar to AP sediments [13], and host a diversity of thermophilic and anaerobic archaeal taxa, phototrophic bacteria, *Firmicutes*, and *Gammaproteobacteria*, whose composition and abundance are mostly dependent on temperature gradients; the presence of *Chloroflexi* as a core microbial group in this hydrothermal system suggests that photoautotrophic carbon fixation is a key process [25]. Comparatively, this phylum is absent in AP sediments, probably as a consequence of the differences in the temperature regimes. Additionally, the pyrosequencing of sediments from saline lakes in Santa Rosa and mats in Laguna Verde, located at over 3700 m of altitude in the Andes Mountains, showed high differences in bacterial diversity and richness [26]; *Bacteroidetes* and *Proteobacteria* phyla were the most abundant and similar in both ecosystems, but sequences for the phylum *Bacteroidetes* were twice as high in Santa Rosa sediments than in Laguna Verde mat samples. Comparatively, the relative abundance of sequences associated with the *Bacteroidia* class was minor in all AP sediments (Figure 3). Although these ponds and the APs showed similar temperatures and pH (7–8), conductivity (5–6 mS cm^−1^) was much lower than in AP waters and sediments, and the nitrate concentration in Santa Rosa (27 mg L^−1^) was nearly 7-fold higher than in the GP and Laguna Verde sediments (4 mg L^−1^) [18,26]. Again, differences in microbial composition are probably influenced by differences in the hydrochemistry of these ecosystems. A similar study compared the composition of microbial communities in the sediments and mats of two geothermal hot springs from Yellowstone and Iceland, finding sequences related to the Archaea and bacteria domains, including many different microorganisms, but a similar community structure in both hot springs [27].

Altogether, the differences in microbial composition among aquatic systems in northern Chile stress the importance of understanding the impact of environmental factors (climate, geology, and hydrogeochemistry) on diversity and adaptive microbial strategies in Atacama. Lakes, high-altitude ponds, and geysers are sources of a rich microbial composition, providing multiple lines of research for microbial ecology and insight on the influence of hydrochemical factors on biodiversity and evolution [23]. In this context, the APs constitute a novel geosite worth exploring due to its microbial diversity. Our metagenomics approach to Amuyo sediments provides novel information on (a) the presence of fungal genus diversity from the phyla *Blastocladiomycota* and *Ascomycota*; (b) virome diversity, particularly from the *Siphoviridae* family and P2-like viruses, opening the possibility to inquire on bacterial and archaeal host assignments and on the presence of haloviruses, among others [28]; and (c) the relatively abundant presence of methanogenic archaea (*Euryarchaeota* phylum) in all AP sediments.

***On the presence of Archaea in the Amuyo ponds***. A phylogenetic study carried out with sinter samples from the El Taito Geyser Field in the Andes highlands demonstrated that *Firmicutes* (*Bacillales* plus *Clostridiales* orders) and *Proteobacteria* phyla accounted for nearly 70% of total bacterial reads, and 22% of the total sequences were assigned to *Actinobacteria*, *Bacteroidetes*, and *Cyanobacteria*, while recovered archaeal sequences comprised less than 2% of total sequences, including *Euryarchaeota* and *Crenarchaeota* (*Halobacteria* and *Thermoproteia*), suggesting the presence of halophilic or methanogenic/methanotrophic archaea [29]. Comparatively, our metagenomic study on AP sediments showed a much lower abundance in archaeal sequences; nonetheless, the genus *Methanosarcina* (*Euryarcheota* phylum) appeared as a major methanogenic member in all pond sediments (Table 2; Figure 3(B.2)). Thus, the environmental conditions of the Amuyo Ponds’ sediments are most probably a better habitat for bacteria, favoring the growth of certain bacterial taxa over archaeal ones. The abundance of methanogens and other archaeal classes indicates that Archaea are present in Amuyo sediments and potentially functioning in these environments. Archaeal methanogenesis occurs in anoxic and reductive environments, and proper substrates for methane production come from the anaerobic degradation of organic matter by an accompanying microflora [31]. We propose that methanogenesis and methanotrophy may be present in the anoxic/oxic layers of Amuyo sediments, since the presence of methanogens and proteobacterial communities enriched in sequences from *Archaeoglobi* and *Halobacteria* was confirmed by our metagenomics data. Thus, the Amuyo Ponds in the Andes highlands would represent a new natural model for methane budget evaluation. Based on 16S rRNA sequences, the *Euryarchaeota* phylum was most abundant in Laguna Tebenquiche, mostly associated with the class *Halobacteria* or anaerobic and methanogenic archaea, suggesting a potential role in methanogenesis in these benthic ecosystems [5].

***On arsenic tolerance***. Isolation, genomic, and biochemical characterization of members from the AP microbiome is needed to advance learning on the diversity and functions of the microbial community in this geosite under particular abiotic conditions. Our metagenomic study showed that AP sediments were enriched in sequences belonging to *Pseudomonas* and *Aeromonas* (Figure 3(A.2)). Sequence analyses allowed us to identify putative genes associated with arsenic resistance in all three AP sediments (Figure 6), suggesting that microbial members of this geothermal ecosystem have developed robust mechanisms to withstand high arsenic content [13,14,15,16,17,18], highlighting the adaptive capability of members of the AP microbiome. Arsenic reductase gene clusters were found in assembled genomes from *Achromobacter*, *Bacillus*, and *Stenotrophomonas* in the YP, *Aeromonas*, *Anaerobacillus*, *Azoarcus*, *Bacillus*, and *Pseudomonas* in the RP, and *Azoarcus*, *Pseudomonas*, and *Thalassospira* in the GP (Figure 7A), including the gene *ars*C (coding for arsenate reductase), *ars*R regulatory genes, and *ars*D, *ars*A, and *ars*B genes, coding for proteins involved in arsenic compound detoxification via a transmembrane efflux channel [32]. Also, the arsenate respiratory reductase (Arr) gene cluster was only detected in *Aeromonas*, *Bacillus*, and *Anaerobacillus* MAGs from Red Pond sediments (Figure 7B); this gene cluster is involved in the reduction of arsenate to more toxic arsenite, and these bacteria may play a critical role in the arsenic biogeochemical cycle, opening potential possibilities to better understand microbial-driven arsenic transformations in this particular ecosystem. The role of microbial communities in modulating arsenic speciation is supported by the presence of the Arsenite oxidase (aox) gene cluster being involved in the oxidation of arsenite to arsenate by *Azoarcus* and *Pseudomonas* in the RP and GP sediments, respectively (Figure 7C). Overall, the ability of these bacteria to carry out both aerobic and anaerobic transformations of arsenic emphasizes their ecological importance and potential use in bioremediation strategies in arsenic-impacted environments. Considering the geographic context, at westbound locations from the Amuyo Ponds, 51 bacteria were isolated from metal-rich sediments at three sites along an east-to-west transect in the Camarones River Basin with a decreasing arsenic content of 498 to 128 mg/kg [32]; these isolates (e.g., *Pseudomonas*, *Aeromonas*, *Sphingomonas*, *Pantoea*, and *Acinetobacter*) were tolerant or resistant to milimolar concentrations of arsenite or arsenate, and some taxonomic similarities and differences can be observed with bacterial members from AP sediments, opening the opportunity for further taxonomic and biochemical inquiries. Finally, the authors inferred that bacterial diversity in the sediments from the sampling sites along the Camarones River was influenced by differences in total organic carbon and arsenic concentration [32].

***On the Amuyo Ponds’ virome***. Compiled information on Atacama Desert viruses has provided insights on their presence, locations, abundance, diversity, and genetic complexity [2,33,34,35,36,37,38]. Metagenomic sequences abundant in mycobacteriophages, gordoniaphages, and streptomycophages in Atacama soils showed a positive correlation with the presence of their corresponding hosts [33]. A mutualistic model has been proposed for host–virus interactions in the driest Atacama core, where microbial cells would provide protection to viruses, which would supply extremotolerance genes to their hosts [35]. It is expected that microbiomes and viromes in highly saline niches contain salt-adapted microorganisms and viruses [2,28]; then, putative cyanophages and *Nanohaloarchaea* viruses were detected in halite samples from Salar Grande, and thirty reconstructed halovirus genomes were lytic viruses, having dominant head–tail structures, resembling findings from other hypersaline environments [35]. Uritskiy et al. [36] reported the presence, diversity, and infective activity of halophilic viruses with transcriptional activity in such salt nodules; the halite virome included members of the families *Myoviridae*, *Siphoviridae*, *Podoviridae* and *Haloviruses*, and the archaeal virus families *Pleolipoviridae* and *Sphaerolipoviridae*. The viral families *Siphoviridae*, *Myoviridae*, and *Podoviridae* were identified in endolithic communities colonizing calcite or ignimbrite, while gypsum was found to bear the *Siphoviridae* and *Myoviridae* families, all showing auxiliary metabolic genes; archaeal Haloviruses were only identified in the ignimbrite metagenomes [37]. Also, novel viral groups with diverse taxonomy were identified in water samples from the Andean Huasco Salar [38].

Based on sequence and phylogenetic analyses, several phage genera were identified in Amuyo sediments, and fourteen viral genomes were assembled (Figure 5, Table 4). Among them, *Bacillus* phage, a tailed, double-stranded DNA virus belonging to the *Myoviridae* family, was identified in YP sediments, infecting members of the class *Bacilli* and phylum *Firmicutes* [39]; bacteriophage Lily (family *Myoviridae*, order *Caudovirales*) and, interestingly, *Rhizobium* phage RR1-B, with *Alphaproteobacteria* as hosts [40], were found in GP sediments; and Circular genetic element sp, a still poorly classified virus species whose hosts include *Proteobacteria*, was present in RP sediments [41]. Then, preliminary evidence exists to show a correlation between Amuyo viruses and the bacterial hosts inhabiting Amuyo sediments; however, further exploration is necessary. In fact, 10 out of 14 assembled virus genomes were not properly identify, nor were their putative hosts inferred (Table 4); this is most probably because the comparison of metagenomic viral sequences with annotated viral sequences, as well as metaviromes from high-altitude wetlands, is limited, as previously commented [38]. Thus, viromes from microbial consortia inhabiting different habitats (endolithic substrates, soils, wetlands, Amuyo sediments), distant emplacements, and different altitudes along the Atacama Desert due to geochemical and climatic influences are now available to be analyzed in terms of their similarities and differences, and Atacama viromes can be explored in search of, among others, lysogenic viruses against pathogenic bacteria to be used as biological controls (e.g., members of the *Podoviridae* family infect *Pseudomonas*, a bacterial genus highly present in Amuyo sediments (Figure 3(A.1))).

## 5. Conclusions

Metagenomic studies show that sediments from the Amuyo Ponds in the Andes highlands in northern Chile are primarily colonized by members of the bacteria domain, with the dominance of *Gammaproteobacteria* (*Pseudomonas*, *Aeromonas*, and *Shewanella*). Most bacterial genera are common in all sediments; however, some are unique, and others are shared among the ponds. The archaeal classes *Archaeoglobi* (phylum *Euryarchaeota*) and Halobacteria (phylum *Euryarchaeota*) in the AP sediments showed enrichment in methanogens (genus *Methanosarcina*). Fungi were mostly represented by the classes *Blastocladiomycetes*, *Eurotiomycetes*, and *Saccharomycetes*. Novel information on the Amuyo Ponds’ metavirome showed differences in composition and diversity, and unclassified phages (*Siphoviridae* family) were most abundant in the YP and GP sediments, with P2-like viruses (family *Myoviridae*) most common in the RP sediments. Putative genes associated with virulence and defense, metal and metalloid tolerance, and secondary metabolites and antibiotics biosynthesis were detected. Metagenomic analyses allowed us to reconstruct 13 bacterial and 14 viral MAGs, most of them with over 90% completion and less than 10% error. Differences in microbial and viral composition and diversity among the Amuyo Ponds’ sediments may be influenced by the chemical contents of the sediments, but further work is needed to support this hypothesis. In summary, the Amuyo Ponds are a novel geothermal geosite; their microbiome and virome have evolved to adapt and bear the local climatic and geohydrochemical conditions.

## Figures and Tables

**Table 1:** Comparative sequencing statistics after metagenomic analyses of Amuyo Ponds’ sediments.

Pond	Sequences Post-QC	GC%	Predicted Function
Green	23,128,825	57 ± 10	8,268,056
Yellow	25,535,805	62 ± 7	7,424,754
Red	27,505,664	57 ± 12	10,157,651

**Table 2:** Domain-related sequence abundance in sediments from Amuyo Ponds.

Domain	Green Pond	Yellow Pond	Red Pond
Bacteria	99.52%	99.71%	99.38%
Archaea	00.08%	00.03%	00.13%
Eukarya	00.28%	00.11%	00.26%
Virus	00.10%	00.11%	00.17%
Others	00.05%	00.01%	00.07%

**Table 3:** Metagenome-assembled bacterial genomes from Amuyo Ponds’ sediments.

Pond	Genus	Completeness (%)	Error (%)
Yellow	*Stenotrophomonas*	100	0
	*Achromobacter*	100	1
	*Pseudomonas*	95	2
	*Bacillus*	98	2
	*Thalassospira*	99	2
Green	*Azoarcus*	100	4
	*Pseudomonas*	100	6.1
	*Aeromonas*	100	5
Red	*Azoarcus*	100	4
	*Anaerobacillus*	92	3
	*Pseudomonas*	91	4
	*Pseudomonas*	99	11
	*Bacillus*	94	16

**Table 4:** Metagenome-assembled viral genomes from Amuyo Ponds’ sediments.

Pond	Virus	Completeness (%)	Error (%)
YP	*Bacillus* phage	101.58	2.07
	NA *	100	4
	NA	100	7.78
	NA	95.82	9.69
	NA	92.68	8.4
GP	NA	102.08	3.78
	Bacteriophage Lily	100	5.98
	NA	100	3.7
	NA	100	2.7
	NA	100	5.57
	*Rhizobium* phage RR1-B	90.21	2.16
RP	Circular genetic element sp	100	8.48
	NA	98.6	9.69
	NA	90.41	1.29

* NA: not assessed.

## Data Availability

Sequencing reads are available at the Sequence Read Archive (SRA) with accession numbers SRR30290574; SRR30505203; SRR30505202.
